# A State-of-the-Art Review of Organic Polymer Modifiers for Slope Eco-Engineering

**DOI:** 10.3390/polym15132878

**Published:** 2023-06-29

**Authors:** Lei Wang, Yongsheng Yao, Jue Li, Kefei Liu, Fei Wu

**Affiliations:** 1 College of Traffic & Transportation, Chongqing Jiaotong University, Chongqing 400074, China; 622210950134@mails.cqjtu.edu.cn (L.W.); yaoyongsheng23@cqjtu.edu.cn (Y.Y.); 2 National & Local Joint Engineering Research Center of Transportation and Civil Engineering Materials, Chongqing Jiaotong University, Chongqing 400074, China; 3 School of Civil Engineering, Central South University of Forestry and Technology, Changsha 410004, China; liukefei92013@163.com; 4 College of Transportation, Jilin University, Changchun 130012, China; wufei1719@mails.jlu.edu.cn

**Keywords:** organic polymers, slope eco-engineering, soil–polymer interaction mechanism, soil stabilization, engineering property

## Abstract

In slope ecological restoration projects, reinforcing soil and promoting vegetation growth are essential measures. Guest soil spraying technology can be used to backfill modified soil and vegetation seeds onto the slope surface, resulting in successful ecological restoration. The use of organic polymer modifiers to reinforce soil has several benefits, such as high strength, effective results, and low pollution levels. Organic polymer soil modifiers can be divided into two categories: synthetic polymer modifiers and biopolymer modifiers. This paper provides a thorough review of the properties and interaction mechanisms of two types of polymer modifiers in soil consolidation. The properties of organic polymer modifiers make them applicable in soil and vegetation engineering on slopes. These modifiers can enhance soil mechanics, infiltration, and erosion resistance and promote vegetation growth. Therefore, the suitability of organic polymer modifiers for soil and vegetation engineering on slopes is demonstrated by their properties and potential for improvement in key areas. Furthermore, challenges and future prospects for slope protection technology using organic polymer modifiers are suggested.

## 1. Introduction

Highway construction inevitably causes disturbance and destruction to the ecological environment of the road area [1]. Therefore, the ecological restoration of highway slopes has been receiving increasing attention. Ecological slope protection technology is a measure that not only enhances the anti-erosion performance of slopes but also simultaneously restores vegetation [2]. Ecological slope protection technology typically includes soil spraying, ecological concrete, geotechnical materials, and biological enzyme protection technology [3,4]. The conventional soil spray seeding remediation technique involves blending backfill soil with humus, plant fiber, chemical fertilizer, and plant seeds before it is sprayed onto the slope to form a conducive layer for vegetation establishment [5]. The technology for the rapid restoration of vegetation on excavated slopes has demonstrated promising ecological and environmental benefits [6,7]. However, there are still some shortcomings that require attention, such as erosion resistance, maintenance costs, and the efficacy of vegetation restoration [8,9,10].

During the initial phase of highway slope protection, engineers primarily focused on ensuring the safety of the slope based on geological structure and hydrological conditions [11,12]. However, ecological engineering and water and soil protection were often overlooked from a comprehensive ecology and environmental protection perspective. As a result, stabilizing and restoring numerous damaged slopes using appropriate restoration measures has become a challenging task [13].

The implementation of ecological slope protection technology involves the utilization of vegetation to reinforce soil and prevent water erosion, resulting in enhanced stability and anti-erosion performance of slopes. Vegetation-based protection for highway slopes entails intercepting rainfall and stabilizing the slope surface through the development of a robust root system [14,15,16]. While vegetation can effectively safeguard slope surfaces, it necessitates high coverage [17,18]. However, the early stages of vegetation growth pose a challenge due to the influence of climate and slope gradient [19]. To ensure successful establishment during this difficult period, engineers have researched various methods, including vegetation concrete and calcium-based soil curing agents such as cement and fly ash [20,21,22]. Despite their effectiveness, these methods do not fully account for the impact of plant growth and soil strength on sloped surfaces [23,24,25]. The introduction of polymer modifiers has opened up new opportunities for ecological engineering on slopes. These modifiers function as both soil stabilizers and plant growth agents, with organic synthetic and biopolymer modifiers being the main categories [26,27]. Organic polymer modifiers have been widely applied in agriculture and industry, demonstrating superior performance compared to traditional ones [28,29]. The environmental benefits of polymer modifiers are undoubtedly noteworthy, particularly in their capacity to mitigate greenhouse gas emissions and minimize natural resource and energy consumption. The current studies suggest that the addition of moderate amounts of polymer modifiers does not have any negative effects. [30,31,32]. When investigating different combinations of organic polymers for slope soil stabilization, it is important to consider potential chemical reactions between them. However, previous research has mainly concentrated on the soil’s curing strength, overlooking its potential effects on vegetation and ecology [33,34,35].

**Figure 1:**
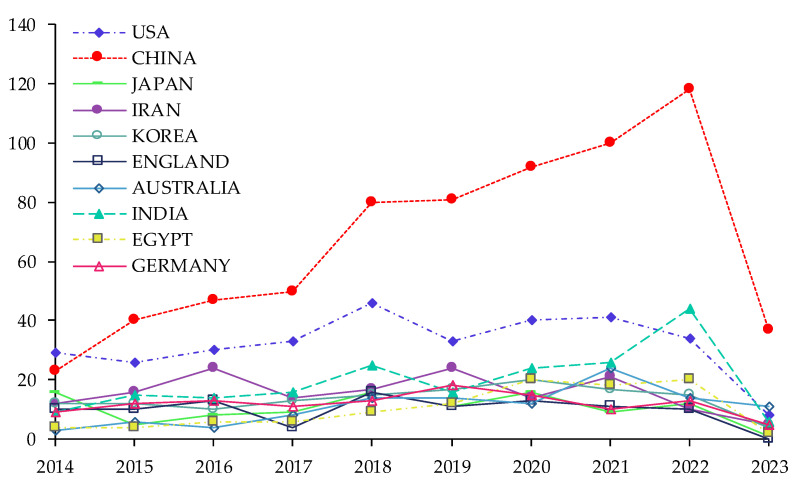
The trend graphs of papers published by ten productive countries.

The authors of this study focused on publications with keywords such as “organic polymers”, “biopolymers”, “soil stabilization”, “slope ecological protection”, “soil erosion”, and “vegetation coverage”. Several scientific databases, including “Web of Science”, “Google Scholar”, “Science Direct”, “Science Citation Index”, and “Scopus”, were used for their search. Figure 1 shows the trend of article publications in ten countries during the past decade. More than 150 works on slope eco-engineering have been reviewed in this study considering different organic polymer types, soil types, vegetation species, interaction mechanisms, mechanical properties, permeability, water retention, erosion resistance, and vegetation growth. The research includes a variety of methods, such as laboratory experiments, microscopic characterization, field trials, monitoring vegetation growth, and evaluating the impacts of various modifiers on soil and vegetation.

This paper provides a comprehensive review of the application of organic synthetic polymer modifiers and biopolymer modifiers in slope soil stabilization and plant growth promotion. The study emphasizes the advantages of using these modifiers over cement and lime, aiming to summarize and discuss their current status, benefits, drawbacks, and future prospects in slope ecological engineering. The authors also examine the fundamental characteristics of organic polymer modifiers utilized in soil stabilization and summarize the interaction mechanism between these modifiers and soil. Furthermore, this research investigates the engineering properties of cured soils modified with organic polymers, including strength, permeability, water retention, erosion resistance, and ability to promote plant growth. Various types of organic polymer modifiers and research approaches are scrutinized. This article highlights research gaps in the current literature and identifies future challenges and directions that require attention. Additionally, this study contributes to the advancement of slope stabilization and eco-engineering applications through the use of organic polymer modifiers.

## 2. The Technology of Ecological Slope Protection

The ecological slope protection technology is based on the principle of vegetation water content and soil consolidation, which utilizes the interaction between plant roots and soil to stabilize rocky and soil slopes. This innovative approach effectively prevents soil erosion, enhances environmental benefits, and mitigates rainwater scouring and erosion on slopes. Vegetation serves two primary functions on sloped surfaces: first, it intercepts and retains rainwater through the dead leaf layer, tree trunks, crowns, and branches above ground; secondly, it forms a mesh structure with tensile and pullout strength via interconnecting plant roots that are widely distributed throughout the soil. This allows vegetation to combine with soil to form a cohesive unit that firmly holds the soil around its root system in place on slopes.

Furthermore, the ecological slope protection technology that employs plant roots must ensure unhindered vegetation growth, with roots growing beneath the shear failure surface to optimize soil consolidation. A suitable soil environment is crucial for promoting plant growth and achieving effective soil stabilization and slope protection. The application of organic polymer modifiers can solidify the soil, facilitating initial plant growth stages and promoting later-stage development. Organic polymer modifiers enhance the mechanical properties, permeability, and erosion resistance of soil. Additionally, biopolymers are abundant in carbon and nitrogen, providing essential nutrients for plant germination and growth.

**Figure 2:**
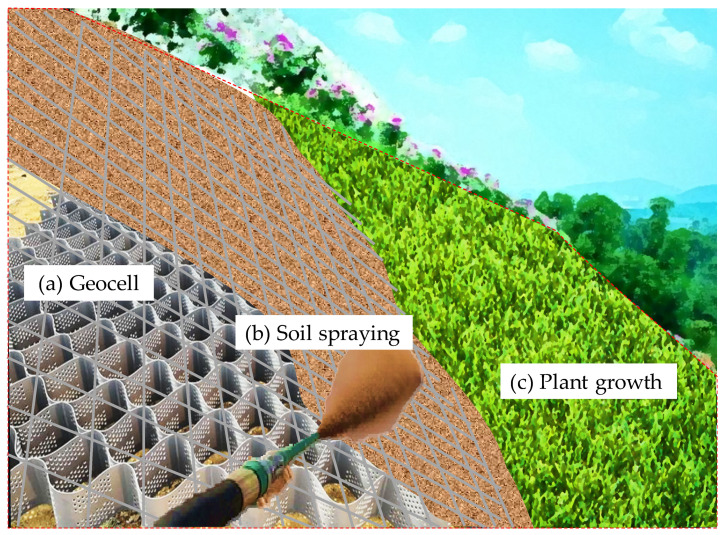
Diagram of soil spraying technology for highway slope stabilization.

The technology of soil stabilization and slope protection with modifiers is mainly realized by spraying and sowing foreign soil. The backfill soil, the modifier, the plant seeds, and other materials are mixed and sprayed on the slope surface to form a soil layer for vegetation establishment. Spray seeding with soil is an effective method to restore the vegetation on the exposed slope, and it has good ecological and environmental benefits for the eroded slope. A diagram depicting the technology of soil spraying for stabilizing highway slopes is presented in Figure 2.

## 3. Physicochemical Properties of Organic Polymer Modifiers

With the development of slope ecological engineering, various types of polymer modifiers are widely used to reinforce soil and promote plant growth. New polymer modifiers for slope ecological restoration are mainly organic synthetic polymer modifiers and biopolymer modifiers. Organic synthetic polymer modifiers are usually artificial polymers consisting of a main chain and side chains. Organic synthetic polymers commonly used in slope ecological engineering include polyacrylamide (PAM), polyurethane (PU), resin, polyacrylates, polyvinyl acetate (PVAc), polyvinyl alcohol (PVA), and methylene diphenyl diisocyanate (MDI). Biopolymers are natural polymers produced by living organisms, such as plants, animals, or microorganisms, and are composed of many interconnected monomers, such as cellulose, lignin, and polysaccharides. Due to the different composition and basic properties of these polymers, they interact with the soil by different mechanisms and have different application effects in slope ecological engineering.

### 3.1. Synthetic Polymer Modifiers

The organic synthetic polymer soil modifier has the advantages of low dosage, stable solidification effect, high plasticity, and good environmental protection, and it can promote plant growth. This modifier mainly comprises polymers generated by artificially controlled polymerization reactions. The common modifiers with major components, such as PVAc, PU, PAM, and polyacrylate, tend to have better corrosion resistance, mold resistance, and high-temperature resistance than biopolymer modifiers.

In ecological engineering applications of slopes, organic synthetic polymer modifiers can promote soil particle agglomeration and improve the resistance to deformation of the soil. Its ecological benefits, i.e., permeability and water retention, should also be considered during slope protection. Most synthetic polymer modifiers are stable and can be used in small concentrations for soil solidification. The composition and basic characteristics of synthetic polymer modifiers are shown in Table 1.

### 3.2. Biopolymer Modifiers

The excretions of animals, plants, and microorganisms produce cross-linked polymers. The gel effect refers to the swelling of biopolymers in soil, which changes soil characteristics and microstructure [42]. It is necessary to research the interaction mechanism of the biopolymer modifier and soil and its characterization and evaluation methods.

Biopolymers are derived from natural resources and are environmentally friendly [43]. Recent studies have shown that biopolymer modifiers are widely used for soil improvement, including soil stabilization [44], strength enhancement [45], and reduction in slope erosion [46]. Biopolymer modifiers are mainly composed of natural metabolites of an organism or an induced microorganism from plant sources, such as agar gum, guar gum, Persian gum, lignin, and starch, from microbial sources, such as xanthan gum, gellan gum, dextran, and β-glucan, or from animal sources, such as casein and gelatin. Biopolymer-based soil treatment (BPST) has been developed to use this modifier to remediate soil [47]. The sources of these common biopolymers for soil stabilization and their basic properties are shown in Table 2.

Agar gum, guar gum, gellan gum, and xanthan gum are all polysaccharides and also widely used biopolymers. Due to their environmental friendliness, these polysaccharides have been widely used in food, medicine, and industry. Lignin is found in high levels in the branches of various trees. Lignin contains hydroxyl hydrophilic functional groups and hydrophobic carbon chains. Lignin by-products are mainly *Lignosulfonate*, kraft lignin, hydrolyzed lignin, and new lignin derivatives, and their characteristics differ. *Lignosulfonate* has many active functional groups, and its solubility is high, while hydrolyzed lignin has poor water solubility and low chemical activity. Recent studies have shown that biopolymer modifiers produced by bacterial and microbial fermentation can be used to stabilize soil. Xanthan gum mainly forms a helical structure and can exhibit high stability over various temperatures, pH values, and electrolyte concentrations [63,64]. β-glucan is found in cellulose and the cell walls of various fungi or bacteria and can be used with other biopolymer modifiers to improve soil properties [65]. Gellan gum is more durable under dry–wet cycles, which is more beneficial for slope ecological engineering in natural environments [55].

## 4. Interaction Mechanism between Polymer Modifiers and Soil

Organic polymer modifiers can form viscoelastic membrane structures with soil, which provides a stable curing effect at low concentrations. The protective measures combined with the organic polymer modifier and the vegetation balance resistance and solidity. The solidified layer can not only prevent the erosion of sand and rain to the lower layer of quicksand but also regulate the water conservation capacity of the sand layer, improve the soil water and heat conditions, and promote the establishment of the soil ecosystem. The ability of soil to fix nitrogen and absorb water is also enhanced, thereby promoting the growth and development of vegetation. Different polymers can treat different types of soil and promote the growth of different plants. Hydrophilic functional groups, such as hydroxyl, carboxyl, amide, and sulfonic acid, have strong adsorption and complexing ability. Water molecules easily enter the internal network, and the molecular chains form a water-blocking layer between soil particles.

### 4.1. Interaction Mechanism between Synthetic Polymer Modifiers and Soil

PAM attaches to clay particles through its molecules and soil cations, such as Ca^2+^, increasing the connectivity of soil pores and improving aggregate stability [66]. Studies have reported that the effectiveness of PAM soil consolidation is related to soil texture and pH value, and the application performance of acidic fine-textured soil is due to alkaline coarse-textured soil [67,68].

PU has stable reaction products, good adhesion, heat resistance, and elastoplasticity when used to reinforce and stabilize the soil. It performs well in slope construction because its short gelation time can reduce the adverse effects of rainfall [69]. The reactive isocyanate groups (-NCO) of PU molecules react with the capillary water in soil particles, forming physical and chemical bonds between PU molecules and soil particles. The PU curing agent is connected with soil particles through chemical bonds to form an elastic membrane structure, thereby enhancing the stability of the soil [70].

PVAc is a transparent amorphous polymer produced by the polymerization of vinyl acetate, which contains many methyl ester groups (-−OOC−CH_3_) on its molecular chain. Additionally, PVAc has good water solubility, is harmless to plant growth, and can significantly improve soil structure, so it is widely used as a stabilizer. Ghasemzadeh et al. [71] studied the microstructural changes in Kaolinite reinforced with a commercial vinyl acetate homopolymer whose main component is PVAc. After the polymer treatment, the soil particles were encapsulated, and a new filler was added between the pores. The polar carboxyl groups on the long chain of PVAc and the clay particles had ion exchange reactions, forming hydrogen bonds or van der Waals forces, wrapping and aggregating the soil particles, and improving the comprehensive properties of the soil. Bu et al. [72] reported that PVAc formed an elastic and cohesive network structure by inter diffusion, infiltration, and entanglement with soil. PVAc can fill soil pores and bond soil particles to each other, thus significantly improving the strength of soil particles.

The main solidification mechanism of PVA is that the polar carboxyl groups on its long-chain macromolecules react with hydroxyl groups or alkali metal ions in the soil, which reduces the thickness of the clay diffusion double layer and increases the bonding between soil particles and aggregate particles [73]. Mirzababaei et al. [74] found that PVA reduced the pore space of the homogeneous matrix of particles, and small agglomerates spaced apart from the pore space gradually connected and formed large agglomerates. The soil stability increased with the increase in curing time. After the polymer solution was dried, a continuous elastic film was formed in the polymer-treated soil matrix. The continuity of the membrane structure made it act as a bridge connecting soil particles [75]. The stabilization and formation of the polymer also depended on the encapsulation of the polymer film, which helped to improve the stability of the aggregate when subjected to external loads or pressures [76]. The pores were filled with the polymer film, thus promoting the formation of micro-agglomerates [77].

Rezaeimalek et al. [78] used a PU precursor with the main component of MDI as a river sand curing agent and reported the encapsulation and filling effect of polymer on sand pore space using SEM.

Hydrophilic groups (e.g., hydroxyl, carboxyl, ether linkage, amino, or amide) in organic synthetic polymer modifiers form a network and produce flocculation in soil particles. The solidification mechanism of organic synthetic polymer modifiers is through bonding and reducing the particle spacing, so their solidification effect on soil depends on the uniform penetration ability of the stabilizer solution to soil particles. Therefore, organic synthetic polymers are more suitable for coarse-grained soils such as sand. For fine clay soil, a large specific surface area will reduce the penetration ability of the stabilizer solution into soil pores.

### 4.2. Interaction Mechanism between Biopolymer Modifiers and Soil

The biopolymer modifier binds to the soil in liquid form and diffuses freely through the macropores within the soil. During the diffusion, the biopolymer first fills the pores inside the soil and continuously wraps the soil particles. The viscosity of the biopolymer modifier makes it aggregate with soil particles to form biopolymer–soil aggregates, thus reducing the intergranular pores and impeding the subsequent expansion of water molecules and biopolymers. Nonetheless, when the concentration of the biopolymer modifier increases to a certain extent, the biopolymer cannot continue to diffuse after the internal pores of the soil are blocked. The results show that the effect of the biopolymer modifier on soil improvement is weakened, and the increase in strength is reduced [79].

With the evaporation of water in the soil, the molecular force between soil particles and the inter-particle bite force increase, exhibiting the improvement of mechanical properties. The biopolymer wraps the soil particles to form polymer chains, which can form a connecting force with the soil particles through hydrogen bonds. Then, the loose soil particles become whole, forming a three-dimensional polymer film and polymer reinforcement chain covering the soil particles. The interaction mechanism between the polymer modifier and the soil is shown in Table 3.

**Figure 3:**
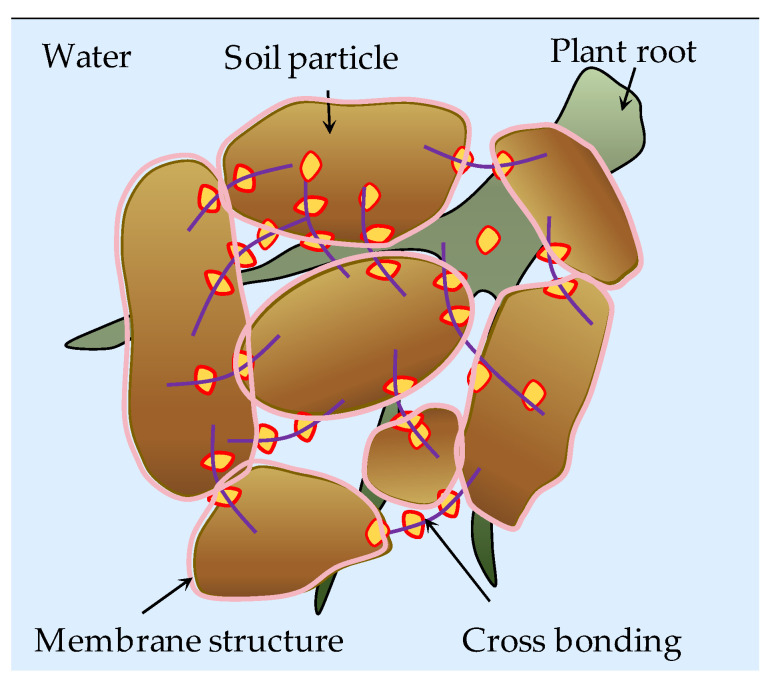
Diagram of the interaction mechanism between polymer modifier and soil.

**Figure 4:**
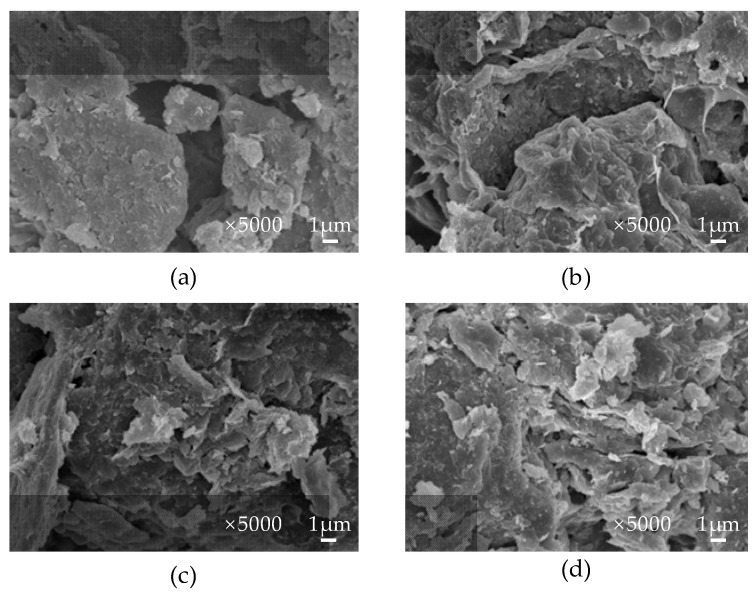
SEM images of soil improved by different polymer modifiers. (**a**) Untreated soil; (**b**) xanthan-gum-treated soil; (**c**) gellan-gum-treated soil; (**d**) guar-gum-treated soil.

The polymer modifiers can improve the strength and the water retention performance of soil, which is the main reason why the polymer modifier can be applied to the ecological protection of the slope. The action mechanism of synthetic polymer and biopolymer modifiers on soil differs. The two action mechanisms are compared, and the results are shown in Table 4. The diagram illustrating the interaction mechanism between polymer modifier and soil is presented in Figure 3. A depiction of the microscopic mechanism behind soil improvement through various polymer modifiers is illustrated in Figure 4.

The synthetic polymer modifier mainly produces an ion exchange reaction between hydrophilic groups (such as hydroxyl, carboxyl, ether bond, amino, or amide) and soil particles to form hydrogen bonds or van der Waals force to wrap and aggregate soil particles and improve the comprehensive performance of soil. Moreover, the modifier fills the soil pores and forms a network structure, and it diffuses, permeates, and entwines in the soil to make its internal structure elastic and viscous. The significant increase in soil strength is attributed to the mutual bonding between soil particles. In addition, the synthetic polymer modifier forms physical and chemical bonds between the molecules and the soil particles, and the modifier and the soil particles are connected through chemical bonds, forming an elastic membrane structure.

In summary, synthetic polymer modifiers solidify soil through particle bonding and spacing reduction. Therefore, their solidification effect on soil depends on the uniform penetration ability of the curing agent solution to soil particles, and those with a higher uniform penetration ability are more suitable for the solidification of coarse-grained soils (sand).

Biopolymer modifiers generate a gel structure in the soil matrix, which exerts coating, bonding, and pore-filling effects on the soil particles, contributing to a compact microstructure and improving the engineering properties of the soil. However, no new minerals are formed, but some functional groups are added. Additionally, biopolymer modifiers are adsorbed on the surface of soil particles through electrostatic action, fill the pores between soil particles, and cement soil particles together to enhance soil stability.

The solution of biopolymer modifiers has a high viscosity, cohesion, and surface tension, and their infiltration ability into soil pores is limited. Therefore, their solidification effect is greatly affected by soil particle size. Biopolymer modifier solution can wrap soil particles by its viscosity and can change the original structure of soil by ion exchange reaction or intermolecular force (hydrogen bond, etc.) with soil particles to reinforce soil.

## 5. Engineering Properties of Polymer-Stabilized Slopes

Polymer modifiers have greatly improved the physical, mechanical, and ecological properties of unsaturated soils. Adding polymer material can promote vegetation growth, and the degradation products are CO_2_ and H_2_O. Therefore, polymer material belongs to ecological soil improvement materials. Soil modifiers are widely used in slope ecological protection or restoration, improving soil shear strength, tensile strength, and erosion resistance and promoting vegetation growth.

### 5.1. Improvement of Mechanical Properties

Due to their special network structure, the polymers greatly enhance the stability of the soil, improve the shear resistance of the root–soil interface, and optimize the tensile strength of the root system. Different organic polymers have been used to stabilize soil with poor structural properties, such as sandy soil with low cementation, loess susceptible to water erosion, and expansive soil prone to expansion and contraction.

Liu et al. [92] investigated the strength and mechanical properties of polymer–root– soil interactions using polyurethane polymers. Different percentages of root content (0, 0.4, 0.8, 1.2, and 1.6% by weight of dry sand) and polymer content (1, 2, and 4% by weight of dry sand) were used. The research shows that the shear stress on the interface is transformed into tensile stress in the root polymer, and the polymer thread and the polymer film form a network structure, which improves the cohesion of the particles. The polymer–root–soil interaction enhanced the shear and tensile strength of the whole structure. With the increase in polyurethane polymer content, the compressive strength of soil was greatly increased, up to 420.94 kPa, and the best effect was achieved at 2–4% concentration.

Huang et al. [93] researched the effects of nano-aqueous adhesive NAA (polyvinyl acetate), (CH2CHCOOCH3)n), on unconfined compressive strength, shear strength, and aggregate characteristics of slope soil. Further, in order to clarify the improvement mechanism, infrared spectroscopy and scanning electron microscopy (SEM) tests showed that NAA was mainly distributed in the interlayer position of flake clay minerals and connected with clay minerals mainly through hydrogen bonding, thus effectively enhancing the cohesion of soil particles.

Liu et al. [82] explored a novel organic polymer soil stabilizer STW (vinyl acetate polymer) and conducted laboratory tests for unconfined compressive strength, shear strength, water stability, and erosion resistance of both STW-treated and untreated soil samples. Field test results showed that stable topsoil had high erosion resistance, and STW significantly affected the surface stability of clay slopes and vegetation growth.

Rezaeimalek et al. [78] used a moisture-activated liquid polymer to solidify sand. The strength of sand increases linearly with polymer addition. The optimum ratio of polymer to water is 2:1. In addition, the unconfined compressive strength of the stabilized sand can reach 5000 kPa, and the samples are stable under both static and dynamic loading.

Sauceda et al. [94] injected polymer into plant roots and measured shear and tensile strengths. The shear strength of polymer-infused roots was increased by 22 kPa (28%) compared with that of original roots; the shear strength of low-plastic clay increased by 13.1 kPa (25%), and the tensile strength increased by 13.6 kPa (55%).

Super-absorbent polymer (SAP) is a kind of environment-friendly soil stabilizer, which can improve the engineering properties of high-absorbent slope soil. Bian et al. [95] studied the unconfined compressive strength and microstructure of SAP-stabilized soil under wetting and drying cycles. SAP can reduce the mass loss of stabilized soil and improve its unconfined compressive strength after dry–wet cycles. After eight rounds of wetting and drying cycles, the strength of SAP-stabilized soil is still 3–4 times that of soil without SAP. In addition, they conducted compression tests for cemented clay with super-absorbent polymer featuring high water contents.

Repeated freeze–thaw cycles will destroy the structure and mechanical properties of soil, further reducing the stability of subgrade slope engineering. The eco-friendly hydrophilic polymer can improve saline soil under freeze–thaw cycles. Reducing the Na+ cation content is an effective way to enhance the performance of saline soil. Xia et al. [96] improved saline soil using hydrosiloxane polymer (also known as hydrogen silicone oil) and investigated the strength characteristics of the treated soil under freeze–thaw cycles. The strength of saline soil treated with polymer increased by 23.1–69.3%, but the effect of inhibiting the adverse effects of freeze–thaw cycles was limited. After 10 freeze–thaw cycles, the strength of soil treated with polymer decreased by 52.7–69.4%.

Bozyigit et al. [97] used xanthan gum, guar gum, and PAM polymer to improve the strength of kaolin under freeze–thaw conditions. Guar gum most remarkably improved soil strength under eight and ten freeze–thaw cycles, and it showed ductile behaviors.

Biopolymer modifiers can effectively improve the compressive strength and shear strength of soil and are widely used in slope ecological protection. Seo et al. [98] mixed a biopolymer binder with site soil and sprayed this mixture to protect the slope. The biopolymer formulation was evaluated by comparing the unconfined compressive strength of the field and laboratory-prepared samples. The biopolymer–soil mixture can reinforce slopes and promote vegetation growth, which, however, requires sufficient cumulative mixing time. Further research is needed to determine the optimal in situ biopolymer–soil mixing formula.

Caballero et al. [99] verified that the 0.5% biopolymer–soil mixture presents the optimum concentration based on its high strength and low swell potential. (With high strength and low swell potential, i.e., 0.5% works best.) The slope strength parameters of unreinforced soil and 0.5% biopolymer–soil mixture are analyzed. Biopolymer-reinforced soil greatly improves the safety factor of shallow slope failure. The long-term properties and durability of biopolymer–soil mixtures still need to be explored.

Acharya et al. [100] conducted a comprehensive study of guar gum biopolymer for repairing the dry cracking of expansive soil slopes. A 3D continuum fast Lagrangian analysis software was used for slope stability analysis, and strength parameters obtained from laboratory tests were also included in determining the range of safety factors of the slope. Furthermore, the biopolymer-treated slope factor of safety was assessed above the acceptable limit.

Fatehi et al. [101] introduced casein and sodium caseinate biopolymers to stabilize sand. Protein-based biopolymers can effectively improve the strength of sand, and the sand treated with sodium caseinate achieves greater compressive strength. Additionally, the curing temperature significantly affects the unconfined compressive strength. When the curing temperature was 60 °C, the soil treated with casein and sodium caseinate exhibited optimum compressive strength. Chang et al. [60] found that the wet unconfined compressive strength of casein–soil mixtures was 480–750 kPa. The improved water resistance means that more effective eco-friendly soil binders can be developed.

Chang et al. [55] investigated the strength-enhancing effect of gellan gum biopolymer on sandy soil under wetting and drying cycles. The strength of sandy soil gradually decreased due to the dissociation of gellan gum monomers under wetting and incomplete recombination during drying. The dry strength of sand treated with gellan gum was still high after 10 wetting and drying cycles. They also studied the interaction between xanthan gum biopolymer and soil. Xanthan gum can enhance the strength of soil particles through hydrogen bonding [102]. The strength obtained by the fine-grained soil comes almost from the hydrogen or electrostatic bonds between the xanthan gum monomers. The results show that the ideal concentration of xanthan gum biopolymer for effective and economical soil improvement is about 1.0% to 1.5%.

Wang et al. [103] carried out wetting–drying cycle tests on the improved soil with 3% lignin content and compared it with the soil improved with quicklime. The stability of lignin-ameliorated soil was better than that of quicklime-ameliorated soil under dry–wet cycles. The results show that the lignin polymer films can encapsulate soil particles and fill internal pores to enhance soil agglomeration.

Rashid et al. [84] have found that the cohesion and internal friction angle, which are parameters related to unconfined compressive strength and direct shear strength, increase with curing time when xanthan gum is mixed with laterite in different proportions. As the pore space is reduced, the soil becomes firmer and denser.

Ojuri et al. [104] used rice husk powder, cassava peel powder, and carboxymethyl cellulose (CMC) as biopolymer-based materials to improve the strength and hydraulic properties of clay. When treated with cassava peel powder (893 kPa) and CMC (450 kPa), the shear strength value of the natural clay (43.5 kPa) increased by 20 and 10 times, respectively.

Ceylan et al. [105] used biofuel by-product (BCP) containing lignin to increase the strength of soil and incorporated 12% BCP in Sandy Silt, which is a powder material with a low lignin content. The research results show that the compressive strength of Sandy Silt increased by four times, and the soil compressive strength increased by two times after adding 12% BCP to Sandy Silt with clay.

Chen et al. [106] explored the influence of xanthan gum biopolymer on the shear strength of sandy soil in the drying process. When the water content is high, xanthan gum biopolymers play a limited role in the sand. In a 40 °C oven, with the continuous evaporation of water, the binding properties of the biopolymer gradually become apparent, resulting in an increase in soil shear strength.

Smitha et al. [107] treated Sabarmati soil with different concentrations of agar biopolymers (0.5, 1.0, 2.0, 3.0%) at different curing times (4 h, 8 h, 1 day, 3 days, 7 days). The shear strength of the Sabarmati soil increased by 384% and 156%, respectively, when 3% and 0.5% agar were cured for 7 days. The treated soil showed a slight decrease in shear strength after soaking but did not affect the soil strength increased by the agar biopolymer.

Khatami et al. [48] mixed 1.0–4.0% agar and 0.5–1.0% starch to improve the compressive strength of sand. The two biopolymers show good compatibility, and the compressive strength of the treated sand changes with polymer content and can reach 4.87 kPa.

This section reviews the research on polymer modifiers for improving soil mechanical properties. Polymer modifiers can effectively improve the compressive strength and shear strength of soil, like geopolymer modifiers. However, the effect on the internal friction angle is limited. Most organic polymer modifiers can improve soil strength in the early stage of maintenance, serving as a beneficial transition phase for slope ecological protection.

### 5.2. Improvement of Permeability

Soil permeability and water-holding capacity are determined by soil mineral composition, pore structure, surface activity of soil particles, and the combination mode of soil particles and water. Soil permeability and water retention determine the ecological performance of the slope and plant growth. Studies have shown that soil permeability decreases immediately after treatment with polymer stabilizers. Single polymers positively affect soil water retention under low suction conditions [108]. However, the soil is in a high suction state under drying-wetting cycles such as rainfall, so it is important to explore the matric suction and water retention characteristics of the soil treated with polymers.

PAM can increase soil water retention capacity and improve soil erosion resistance. Sepaskhah et al. [109] investigated the effects of PAM proportions on runoff, soil erosion, and water infiltration. Wastewater has a greater ability to resist soil erosion and maintain slope stability than freshwater.

Nevertheless, studies have shown that monomeric PAM has some genotoxicity [110], while excessive PAM can lead to the plugging of soil porosity, thus decreasing soil permeability and increasing the runoff rate [111]. The biodegradation process of PAM and its effect on the soil microbial community are unclear. Ma et al. [112] evaluated the degradation efficiency of *Klebsiella* sp. PCX–biochar composite for PAM in soil, and *Klebsiella* sp. PCX was used to remove PAM from the soil by loading biochar with the bacteria. Given the poor effect of polymers alone and their side effects, most SAPs are composite polymers, i.e., a combination of multiple polymers.

Yu et al. [113] conducted a series of tests to analyze the water retention properties of mixtures by using different SAPs in different soils. The SAP with the highest charge density (GNKH) absorbed the most water and maintained the highest water content during the drying for up to 7 h. However, excessive SAP can cause complete blockage of soil voids, preventing effective infiltration. In contrast, an appropriate amount of SAP can significantly reduce water permeability through the soil profile. Through experiments, Misiewicz et al. [114] revealed that the size of SAP particles is an important parameter for the application to different types of soil.

Huang et al. [115] developed a novel polymer composite, namely water-dispersed nano-adhesive (ADNB), composed of different proportions of polymer binders and resins. ADNB not only improved the early strength and erosion resistance of the slope but also changed the soil structure and increased the porosity and water-holding capacity, thus improving the ecological self-healing ability of soil. After treatment, the soil’s effective water content increased by a range of 0.72% to 9.26%. In addition, a prerequisite for soil improvement is the optimal amount of resin. The long-chain polymer molecules of the resin can absorb and store large amounts of water by binding to water molecules. When the resin concentration was at 0.01% and 0.015%, there was an initial increase in plant root biomass followed by a subsequent decrease as polymer concentration increased. After the hydrophilic groups of PVAc are combined with the soil aggregates, the polymer binder wraps the soil particles and interconnects them to form a film structure with viscosity and elasticity. It is difficult for these membranes to absorb water, but they can change the void structure in the soil and affect the formation of a honeycomb structure in the solidified layer. The infiltration rate of water is accelerated, and the evaporation of water is suppressed.

Huang et al. [93] explored the water-holding capacity of super-absorbent resin (SAR) in slope topsoil. The volume expansion and contraction of SAR during water absorption and drainage loosened soil and improved the soil microstructure. The percentages of soil aggregates (particle size ≥0.25 mm) containing SAR levels ranging from 0 to 1% were 59.9%, 83.9%, 93.3%, 98.8%, and 98%. The strong water absorption and retention capacity of SAR improved the water-holding capacity of the soil.

**Figure 5:**
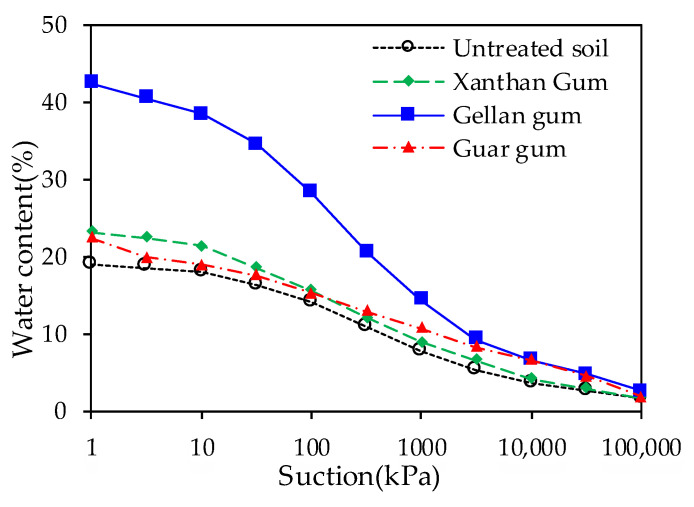
Water retention characteristics of soil improved by various biopolymer modifiers.

Chang et al. [116] evaluated the effect of xanthan gum biopolymers on the wetting and drying processes of the soil through Fredlund-type soil water characteristic curves. Xanthan gum significantly decreased the capillary conductivity of the soil, and the capillary conductivity decreased at a rate of about 10^−7^ m/s after the treatment with 1.0% xanthan gum. The water retention characteristics of soil improved by various biopolymer modifiers are illustrated in Figure 5.

Biju et al. [117] investigated the permeability of a biopolymer-modified sand–bentonite mixture (SBM), a biopolymer obtained by mixing guar gum and xanthan gum. The biopolymer remediation agent-induced aggregation of clay platelets, which also created a wider effective flow path. The leaching of leachate caused the diffusion bilayer of the clay surface to shrink and increased soil porosity.

The soil–water retention curve (SWRC) can describe the relationship between soil gravity, volumetric water content, and soil suction. It can also reflect the permeability of unsaturated soil [118]. Based on the Fredlund–Xing equation, Gao et al. [119] developed a new water retention model, which can well explore the effect of initial porosity on SWRC under wetting and drying conditions.

The soil water retention curve (SWRC) is one of the most important hydrological parameters in seepage analysis. It studies the relationship between soil suction and water status. The water retention behavior of biopolymers in the soil is very complex due to the change in soil microstructure. Rosenzweig et al. [120] investigated the SWRC of the soil and xanthan gum–soil mixtures. The enhanced water-holding capacity of xanthan gum improved the water-holding capacity of the soil–xanthan gum mixture (relative to pure soil). The addition of xanthan gum changed the pore size distribution and WRC of the soil due to occupying pores or expanding pore space. The pure xanthan gum WRC was determined by equilibrating xanthan gum samples stored in dialysis bags with different polyethylene glycol (PEG) solutions [59]. According to the relationship proposed by Michel [121], solutions of defined water potentials were prepared by varying the PEG 8000 (Union Carbide, Danbury, CT, USA) concentration.

(1) $$P=0.129{\left(C_{\mathrm{PEG}}\right)}^{2}T-14{\left(C_{\mathrm{PEG}}\right)}^{2}-0.4(C_{\mathrm{PEG}})$$

where *P* is the water potential (MPa), *T* is the temperature (°C), and *C*_PEG_ is the PEG concentration (kg PEG/kg water).

Zhou et al. [122] proposed a new SWRC model for soil amelioration by biopolymers. The gravimetric water content *w*(gg^−1^) of the biopolymer-modified soil, which was defined as the ratio of the mass of pore water to the total mass of the solid components, including all soil particles and biopolymers, as shown in the equation.

(2) $$w=\frac{f_{w(s)}+f_{p}f_{w(p)}}{1+f_{p}}$$

(3) $$f_{w(p)}^{*}=\frac{a}{1+s^{b}}$$

(4) $$C_{in}=\frac{1}{1+{\left(e_{w(p)}^{*}/e_{0}\right)}^{c}}$$

(5) $$f_{w(p)}=f_{w(p)}^{*}C_{in}$$

(6) $$f_{w(s)}={\left(1+{\left(\frac{s{\left(e_{0}{}^{2}/(e_{w(p)}+e_{0})\right)}^{m_{4}}}{m_{3}}\right)}^{m_{2}}\right)}^{-m_{1}}\frac{e_{0}{}^{2}}{(e_{w(p)}+e_{0})G_{s}}$$

(7) $$w=\frac{{\left(1+\left(\frac{s{\left(e_{0}{}^{2}/(e_{w(p)}+e_{0})\right)}^{m_{4}}}{m_{3}}\right)m_{2}\right)}^{-m_{1}}\frac{e_{0}{}^{2}}{(e_{w(p)}+e_{0})G_{s}}+f_{p}\frac{a}{1+s^{b}}\frac{1}{1+{\left(e^{*}/e_{0}\right)}^{c}}}{1+f_{p}}$$

where *w* is the gravimetric water content; *s* is soil suction; *f_p_* is the biopolymer content in soil; *f_w(p)_* is the ratio of water mass in the first form to the mass of biopolymers; *f_w(s)_* is the ratio of water mass in the second term to the mass of soil particles; f_w(p)_* and e_w(p)_* are the maximum values of *f_w(p)_* and *e_w(p)_*, respectively; *a* and *b* are positive model parameters, controlling the saturated water content and desorption rate, respectively; *c* is a positive soil parameter; a new variable *C_in_* is proposed to describe biopolymer–soil interactions; *e_0_* is the initial value of the effective void ratio prior to biopolymer swelling; *e_w(p)_* and *e_w(s)_* are the ratios of water volume in the first and second forms to the volume of soil particles, respectively; *m*_1_, *m*_2_, *m*_3_, and *m*_4_ are positive soil parameters; *G_s_* is the specific gravity of soil particles, which is defined as the ratio of soil particle density to water density.

(8) $$e_{p}=V_{p}/V_{s}$$

(9) $$e_{w(p)}=e_{w(p)}^{*}C_{in}$$

(10) $$\Delta e=\frac{e_{w(p)}{}^{2}}{e_{w(p)}+e_{0}}$$

(11) $$\theta =\frac{w(1+f_{p})G_{s}}{1+e_{p}+e_{0}+\Delta e}$$

(12) $$S_{r}=\frac{w(1+f_{p})G_{s}}{e_{0}+\Delta e}$$

where *e_p_* is the volume ratio of dry biopolymers and soil particles; vs. is the total volume of all soil particles; *V_p_* is the total volume of all biopolymers in a completely dry state; Δ*e* is the incremental void ratio induced by the swelling of soil; *θ* is the volumetric water content; *S_r_* is the degree of saturation.

Equations (7), (11), and (12) can be used to calculate the gravimetric water content–suction relation, volumetric water content–suction relation, and saturation–suction relation, respectively. For the new SWRC of biopolymers, the reliability of the model was verified through experiments. Biopolymers can occupy part of the pore space of the soil and reduce its water retention capacity. The water absorption of the biopolymers is reduced due to the limitation of soil particles compared with that of pure biopolymers [49,123,124,125].

Huang et al. [126] developed an empirical model to describe the effect of polymers on the soil–water characteristic curve based on experimental data. The effects of nano-aqueous binder (NAB) and super-absorbent resin (SARn) on soil water properties and pore size distribution were investigated. The amended soil showed a significant increase of 6.09% to 16.54% in cumulative pore volume and up to 16.79% increase in saturated water content compared to natural soil. The addition of composite polymers has been found to enhance soil water retention capacity by quantifying water uptake and storage. Furthermore, these polymers can effectively reduce the number of pores with diameters of 1.11–8.3 μm, increase the number of pores with diameters of 0.5–1.11 μm, and have little effect on the number of pores with diameters of 0–0.5 μm.

At present, organic synthetic polymers and composite polymers are commonly used to improve the permeability and water retention of the soil. These composites contribute to soil self-healing by altering its structure, pore change, and infiltration rate. Nonetheless, the effect of biopolymers on soil permeability behavior has rarely been reported. Few studies have accurately measured the permeability of soils enhanced by biopolymers. Soil moisture is affected by various factors, and there is a lack of research on the mechanism of soil water retention improvement by curing agents [127]. In the present study, the influence of different soil types on SWCC under different conditions (such as dry–wet cycles and freeze–thaw cycles) is considered. Nevertheless, little has been reported on the SWRC of organic polymer-modified soil, especially the water retention mechanism after polymer treatment.

### 5.3. Improvement of Erosion Resistance

**Figure 6:**
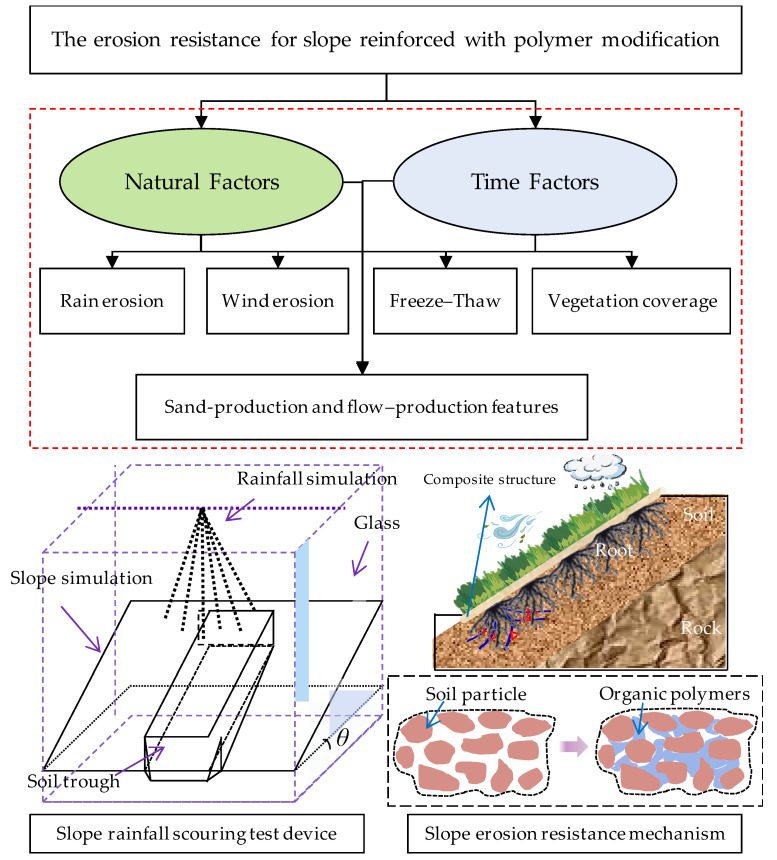
The erosion resistance test flow chart for a slope reinforced with polymer modification.

Soil and water loss involves many ecological and environmental problems in geotechnical engineering, slope ecological protection, road engineering, reservoir bank slope protection, etc. Soil and water loss hinder the growth of slope vegetation. Slope soil erosion can be described as the separation, migration, and deposition of surface soil on slopes. Soil erosion rate refers to the mass fraction of the eroded soil mass in the original soil mass, which can well reflect the erosion resistance performance of the soil. Many indoor rainfall simulation experiments have shown that a small amount of stabilizer can significantly improve soil resistance to hydraulic erosion. The erosion resistance test flow chart for a slope reinforced with polymer modification is depicted in Figure 6.

Continuous rainfall and rainstorms can lead to soil erosion and runoff, and the interfacial shear stress caused by water flow can cause surface soil particles on slopes to peel off. Soil erosion on slopes not only affects the growth of vegetation but also causes significant soil erosion. Therefore, improving the performance of topsoil is the key to preventing water and soil erosion.

Through laboratory tests, Liu et al. [18] investigated the effects of PU concentration on the strength, permeability, water retention stability, water retention capacity, and erosion resistance of treated sands. The results showed that the erosion behavior of topsoil can be changed to the greatest extent by controlling the PU concentration below 10%.

Chen et al. [128] explored the effects of PAM with different formulations on soil nutrient loss and soil erosion by simulated rainfall experiments. Nitrogen and phosphorus are key trace elements for vegetation growth on slopes. The use of PAM resulted in a reduction in total nitrogen loss by 35.3–50.0% in comparison to the control group. It was observed that particulate-bound nitrogen and phosphorus were the main contributors to nitrogen and phosphorus loss during runoff. Post PAM treatment, water-stable aggregates increased by 32.3% to 59.1%, total porosity increased by 11.3% to 49.0%, final permeability increased by 41.3% to 72.5%, and soil erosion decreased by 18.9% to 39.8%.

Levy et al. [129] and Al-Abed et al. [130] added a small amount of PAM to the soil to reduce soil loss caused by rill erosion. Inbar et al. [131] explored the mechanism of soil infiltration rate (IR), runoff, and soil loss changed by anionic PAM. Lu et al. [132] compared the effects of PAM and a polysaccharide (Jag C 162) polymer as soil amendments on reducing soil erosion. In contrast, JagC 162 showed a more pronounced effect than PAM in reducing erosion.

Chang et al. [17] conducted a study on β-glucan and xanthan gum to improve the resistance of red loess to water erosion. Under long-term water erosion, the soil erosion rate decreased from more than 30% to less than 2%. Additionally, the combination of slope vegetation measures and soil compaction improvement significantly enhanced soil erosion resistance. Jr et al. [133] used xanthan gum to reinforce silty sand soil and studied the wind erosion resistance of the stabilized soil using a small wind erosion model box. The results showed that spraying xanthan gum could significantly reduce the wind erosion rate of the soil, and xanthan gum with a higher concentration had a better effect.

Biopolymer-based soil treatment (BPST) is a novel ground improvement method using organic biopolymers as novel soil binders. Chang et al. [134] reviewed the application of BPST in soil improvement and erosion control in geotechnical engineering. Different biopolymers are practiced differently in this field, but most can improve soil erosion well. Biopolymers have certain economic feasibility and sustainability in soil stabilization and slope protection.

In addition to being derived from animals and plants, biopolymers can also be produced from microorganisms, known as microbial biopolymers. Ham et al. [135] reported the role of microbial biopolymers in soil erosion resistance. *Leuconostoc mesenteroides* were cultured and stimulated in fine sand to produce insoluble polysaccharide biopolymers called glucans. The combination of microbial biopolymers with soil increases the critical shear stress and surface erosion resistance of soil by enhancing the cohesion of the particle-coated biopolymer slime and reducing seepage through pore plugging.

In addition, the composite polymer markedly improves the erosion resistance of the slope. Cao et al. [136] explored the role of SAPs in soil erosion through artificial rainfall experiments. The results show that the surface runoff and sediment are remarkably reduced. The addition of SAPs resulted in a significant reduction effect on runoff, ranging from 24.6% to 60.6%. Moreover, the soil water content was improved by over 30% compared to the control group.

Yang et al. [137] prepared a new soil stabilizer, modified carboxymethyl cellulose (M-CMC), from CMC and PAM. The effects of 0–1.3% M-CMC on the shear strength, permeability, water sensitivity, and microstructure of silty sand were investigated by indoor and outdoor tests. Rainfall simulation tests show that M-CMC can effectively reduce the infiltration rate and improve the erosion resistance of the slope.

Liu et al. [138] prepared S-type and E-type soil stabilizers by using acrylate monomer and acetic-ethylene-ester polymer, respectively. Soil water stability was evaluated based on the index K, which was calculated by identifying the mechanism of physicochemical bonding to form a film structure on the surface of aggregates. The effect of a low concentration of E-type (K = 90.1% at 3%) was better than that of a high concentration of S-type (83.8% at 40%), indicating that the film structure formed by polymers on the surface of aggregates could reduce soil erosion.

Sun et al. [139] added polyacrylamide (PAM) to the cementing agent and explored the surface erosion resistance of loess slopes by microbially induced calcite precipitation (MICP) in combination with PAM. The test results showed that adding 1.5 g/L of PAM achieved the best erosion control and surface strength. This was mainly attributed to the fact that PAM enhanced the tensile and shear strength of the loess, and MICP-PAM provided a stable precipitation spatial structure. Zhao et al. [140] used polyacrylic acid (PAA) working in concert with enzyme-induced carbonate precipitation (EICP) to improve the strength of loose soils. It was found that PAA could extend water supply time and improve soil strength for EICP, mainly in terms of surface soil crust stiffness and thickness, which significantly affected soil stabilization and erosion resistance. Kebede et al. [141] explored the potential of anionic PAM to reduce soil erosion through soil conditioning.

Additionally, this study compared PAM in combination with lime versus alone, and the greatest reduction in soil erosion was observed with PAM in combination with lime and PAM alone. PAM binds fine soil particles together to reduce particle floating and increase their deposition rate, resulting in the higher stability of soil aggregates [142]. Sadeghi et al. [143] investigated the potential of biochar and PAM alone and in combination with lime to reduce soil erosion. Sadeghi et al. investigated the effect of biochar and PAM application alone and in combination on the erosion variables of loess and marl soils.

Zezin et al. [144] developed interpolyelectrolyte complexes by interacting with oppositely charged polyelectrolytes. The composites form a soil polymer crust to prevent wind and water erosion. The crust can self-heal after rain damage, and the self-healing properties are attributed to the reversibility of electrostatic interaction.

Most of these studies focus on the erosion process of slope soil, and the anti-erosion properties of various new composite polymers acting on slope soil are rarely investigated. The anti-erosion performance of the polymer-stabilized soil is improved, but the related mechanism needs to be further explored.

### 5.4. Promotion of Vegetation Growth

Plant growth and root system development are crucial in ecological slope protection engineering. Because of the influence of climate environment and slope gradient, the plant slides down along the slope before it is rooted. Enhancing early vegetation growth and improving soil strength is one of the prerequisite factors for ecological slope protection. Existing studies have shown that some polymer stabilizers can promote plant growth by changing soil structure, reducing soil erosion, and promoting soil water retention. The research progress on polymer curing agents for promoting plant growth is shown in Table 5.

It can be summarized from Table 5 that PVAc, B-glucan, and XG could promote seed germination and seedling growth. These stabilizers could affect the water retention capacity and structure of soil and plant root activity by regulating the content of organic matter in the soil and further impact plant germination and growth.

The network gel structure formed by curing agents has good water absorption capacity, and when the soil moisture content is low, the gel structure releases water to soil particles through osmosis, improving the water utilization rate of plants. Yang et al. [137] found that the amount of soil loss and the number of gullies decreased and the vegetation coverage increased after sand slope stabilization by M-CMC. In the slope ecological restoration project, the loose soil on the surface is prone to erosion by water flow during watering maintenance, causing the ungerminated grass seeds to be exposed and taken away by the water flow and further degrading the restoration efforts. Spraying curing agents can solve this problem. Liu et al. [82] conducted field tests and showed that spraying STW stabilizers can reduce slope soil erosion, improve seed survival, and increase vegetation coverage. Regarding the composite application of curing agents, Huang et al. [152] found that nano-aqueous binders filled the pores and thus improved the soil microstructure; these binders also increased soil available water content and raised seed germination rate and biomass.

A higher PVA content contributes to a better water-holding capacity and plant growth performance. The unique molecular structure of PVA facilitates its interaction with water molecules, enabling the absorption of a substantial quantity of water and water storage in long polymer chains [153]. Therefore, PVA exhibits good water absorption and instantaneous water-holding capacity [154]. The hydrogen bond in water combines with the hydroxyl group in the polymer, lowering the soil evaporation rate and providing water necessary for the plant. Nutrient supply is also a key factor influencing the growth of plants. It has been reported that PVA can enhance the ability of plants to absorb nitrogen [155]. This explains the better growth of plants treated with PVA. When the content of PVA exceeds the defined range of 3%, these beneficial effects are weakened, possibly due to the limitation of the water-holding capacity of PVA. PVA can also improve the soil structure and porosity, thereby reducing the heat exchange between air and soil and providing favorable conditions for plant growth [115].

Li et al. [156] developed an environment-friendly polymer compound fertilizer (PCF). The slope planting test showed that the PCF could significantly promote plant growth. After PCF treatment, the germination rate of the plant increased from 31% to 68%, and the survival rate increased from 45.2% to 67.7%. The PCF enhances the growth of slope vegetation in arid and semi-arid areas and meets the needs of slope protection and soil and water conservation.

Huang et al. [157] conducted a long-term monitoring experiment on ADNB to promote plant growth. It has advantages in promoting shrub growth, but as time increases, the growth rate of the plants will slow down. Compared with natural slopes, the germination rate of herbaceous plants treated with ADNB increased by 11–36%, the germination time was advanced by 40–60%, and the plant height was increased by 12.9–100%. The germination rate of shrubs increased by 2.3–18.1%, the germination time advanced by 37.5–53.8%, and the plant height increased by 28.6–168.8%.

Zhang et al. [158] studied the effects of bio-fertilizers and SAPs on plant growth. The application of bio-fertilizers and SAPs decreased the pH value and increased the available NPK and soil organic matter significantly.

Yang et al. [159] explored the soil improvement effect of SAPs, and the indexes, including saturated water absorption, evaporation rate, water-holding capacity, plant germination rate, and survival rate in the improved soil, were evaluated. The results showed that the polymers improved soil physical properties and promoted vegetation growth and development. The effects of SAP content (0.15%, 0.30%, and 0.45%) on seed germination were researched, and it was found that the optimum content was 0.30%.

Su et al. [145] selected PAM, sodium polyacrylate, Balite™ efficient poly agent (Heze Tianling Agrochemicals Co., Ltd., Shandong, China), Hankeshu™ aquasorb (Beijing Sangsong Eco-Technology Co., Ltd., Beijing, China), and Zhengyuan™ aquasorb (Henan Zhengyuan Bio-technology Co., Ltd., Henan, China) as water-absorbent materials to improve the emergence rate and survival rate of seeds. *Caragana korshinskii* seeds with added SAPs had a high emergence rate, and Hankeshu™ aquasorb, whose active ingredients are acrylamide and potassium acrylate copolymer, exerted the most remarkable effect. The second most effective absorbent is Zhengyuan™ aquasorb, which contains acrylamide and sodium acrylate copolymer.

**Figure 7:**
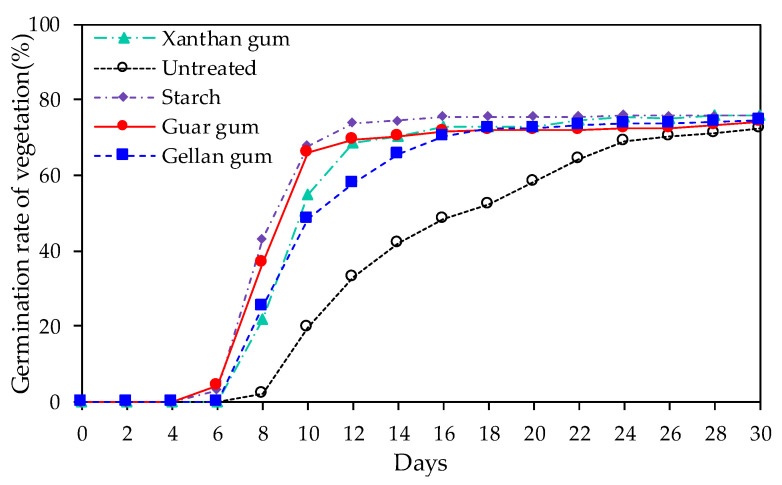
Germination rate of vegetation after treatment with different polymer modifiers.

Wang et al. [83] explored the effects of Xanthan gum, gellan gum, and guar gum on the water retention characteristics of silt soils and the promotion of vegetation growth. The research results indicate that gellan gum has better water retention performance in silt soils. In addition, the soil germination rate and vegetation growth height after treatment with xanthan gum were higher, mainly attributed to the hydrophilicity and adhesiveness of the polymer. The germination rate of vegetation after treatment with different polymer modifiers is illustrated in Figure 7.

Biochar is widely used as a soil conditioner [160]. It can increase soil water retention capacity [161], improve soil quality and fertility [162], provide plant nutrients, and promote plant growth [163]. Biochar can also reduce runoff and soil erosion [164]. However, it has been reported that biochar will slide down the slope with the exfoliated sediment due to rainfall runoff and erosion [165]. The main reason for this is that biochar has poor adhesion properties and will float in the water. When high molecular polymers are used in combination with biochar, the biochar particles are stabilized by binding them together and with soil aggregates for a long time. Polymers bind soil particles together and interact with biochar to mitigate soil erosion, especially in loose soils [166].

In most studies on promoting plant growth, composite polymer curing agents were used, possibly due to the limited efficacy of single organic polymers. In addition, if it is desired to achieve rapid growth and high vegetation coverage on the slope, the curing agents need to meet multiple conditions. Moreover, the “matching degree” of plant species and curing agents also affects plant growth and development. From this literature review, it can be summarized that there are few studies on using biopolymers to promote plant growth. Future studies should compare the capacity of synthetic polymers and biopolymers to promote plant growth.

## 6. Challenges and Future Prospects

This review highlights certain unresolved challenges in the application of organic polymer modifiers for slope eco-engineering which require further research to enhance their effectiveness. Future developments in slope protection technology using organic polymer modifiers should prioritize addressing the following deficiencies:

- Organic polymer materials have the potential to replace traditional technologies used to protect highway slopes due to their renewable and sustainable nature. The cost of biopolymers has decreased by over 80% between 1990 and 2014, making large-scale production and application more cost-effective. In addition, incorporating biopolymer modifiers with a mass fraction between 0.2% and 0.5% in soil reinforcement and vegetation growth promotion processes can significantly reduce material costs compared to synthetic organic polymers. Although the biopolymer industry is still in its developmental stage, its economic feasibility is expected to improve over time.
- The preparation of biopolymer modifiers presents a significant challenge, particularly given the high purity standards required for use in the food and medical sectors. As a result, production costs are currently high. However, if purity standards were relaxed, production expenses could be halved. While biopolymers are primarily used in the food and medical sectors, there is a growing demand for their use in slope engineering, where technical requirements are lower. This increased demand is expected to drive improvements in biopolymer synthesis technology.
- The use of biopolymer modifiers in ecological slope restoration is crucial for mitigating climate change and promoting ecological health. Biopolymers, in particular, offer superior environmental properties and benefits compared to traditional curing materials. By reducing CO_2_ emissions from synthetic sources, biopolymers can address the negative impact of cement production on the environment. Currently, cement is the most commonly used curing agent for reinforced soils, but it generates approximately 1 ton of CO_2_ per ton of cement produced. By adopting biopolymers as an alternative, it can significantly reduce this environmental impact.
- There is a lack of research on the carbon sequestration potential of vegetation on roadside slopes, and there is a need for systematic and quantitative estimation studies. However, the ecological engineering of roadside slope vegetation can effectively utilize plant photosynthesis to absorb CO_2_ emitted by vehicle exhausts. The decarbonization of organic polymers is currently a priority in ecology, and further research should be conducted to explore the potential of decarbonization in both organic polymers and vegetation.
- Most previous research is conducted through laboratory macro- and micro-experiments. Future research on organic synthetic and biopolymer modifiers should be performed in complex natural environments. It is necessary to carry out large-scale slope ecological protection and outdoor tests using organic polymers. This is a prerequisite for the extensive application of these modifiers.
- Considering the rainfall in natural environments, whether the organic polymer modifier can maintain its effect under dry–wet cycles and continuous rainfall needs further study. The durability of organic polymer modifiers in a natural environment still needs to be tested to ensure that the vegetation has been established on the slope. Under the initial protection of organic polymer modifiers, a “protective cover” is provided for vegetation growth, and the slope surface is reinforced. The durability improvement can be accomplished by combining various types of polymers or by designing new polymers.
- A wide variety of polymer modifiers can promote plant growth, but few studies have considered the toxicity of these polymers or composite polymers. It is not clear whether these polymers pollute the surface soil and slope groundwater. Therefore, it is necessary to monitor polymers and plants to evaluate their environmental performance. Additionally, the current research on promoting plant growth is mostly a short-term (about one month) observation, while slope vegetation protection is a long-term task. Therefore, the growth status of vegetation should be continuously tracked and monitored in the future.
- The soil erosion model can estimate runoff and erosion levels at different points in a slope watershed. It considers factors leading to erosion and sediment yield, including rainfall, interception, surface water flow, and sediment transport. The erosion degree of soil solidified by polymer modifiers is mainly evaluated based on runoff, and the erosion mitigation mechanism of polymer modifiers is ignored. Furthermore, the new soil erosion model after polymer modifier solidification needs further investigation for more effective soil erosion control by polymer modifiers.

## 7. Conclusions

Increasing environmental awareness and fast-paced societal development have led to the widespread use of organic polymer technology for ecological slope protection in sustainable infrastructure development. This study summarizes the use of organic synthetic polymer and biopolymer modifiers to promote soil consolidation and slope vegetation growth. The focus is on the fundamental characteristics of organic polymer modifiers, their interaction mechanism with soil, and the engineering and ecological properties of stabilized soil. The current state of the art for mechanical properties, infiltration properties, erosion resistance, and vegetation growth of organic polymers was reviewed. Furthermore, the significance of organic polymers in ecological engineering for road slopes was introduced, and their advantages in achieving sustainable objectives in infrastructure development were highlighted. Organic polymer enhancement techniques have been used alongside traditional slope protection methods, but there are technical challenges to this approach. Further research is needed to fully understand these mechanical mechanisms of composite slope structure and improve its overall performance. This will lead to the development of standardized testing procedures and design methods for organic polymer-modified slopes in the future.

## Figures and Tables

**Table 1:** Composition of organic synthetic polymer modifiers for soil curing and their basic properties.

Polymer Modifier Types	Composition	Basic Features	Reference
PAM	Synthesized from acrylamide subunits with straight chain or cross-linked conformation.	Cross-linked PAMs are water-absorbent and insoluble, but linear-chained PAMs are water-soluble.	[36]
		Linear-chained PAMs are suitable for soil reinforcement.	
PU	Composed of macromolecular polyols, polyisocyanates, etc.	The reaction products are stable, with good adhesion, heat resistance, and elasticity and short gelation time.	[37]
Polyacrylate	Consists of monomers of acrylic acid and esters.	Easily polymerizes with other functional groups to form different polymers.	[38]
		Good hydrophilicity and high reactivity with vinyl and carboxyl groups.	
PVAc	Synthesis from vinyl acetate monomer.	Insoluble in water but soluble in benzene, acetone, etc.	[39]
		Good adhesion.	
PVA	Prepared by alcoholysis of poly (vinyl ester).	It has water solubility and is highly polar.	[40]
MDI	Condensation of aniline with formaldehyde, followed by reaction with phosgene.	Its NCO group reacts easily with OH groups in water to form a mixture of diisocyanates and amines, and the solid mixture binds the soil particles together.	[41]

**Table 2:** Composition of biopolymer modifiers for soil curing and their basic properties.

Polymer Modifier Types	Source	Basic Features	Reference
Agar Gum	*Rhodophyta* (Red algae).	Belongs to reversible gels, i.e., it can be dissolved in boiling water and forms a gel after cooling at about 35 °C.	[48,49]
		Agar gels have rheological properties, hydrophilic.	
Guar Gum	*Cyamopsis tetragonoloba* (Leguminous shrub).	Rapid hydration in cold water, high-viscosity solutions can be formed even at low concentrations.	[50,51]
		Natural decomposition to monosaccharides and water by the action of microorganisms or enzymes, extreme pH, and temperature degradation.	
Persian gum	The trunk and branches of wild almond trees of Zagros forests in Iran.	Anionic polysaccharide, a plant exudate gel.	[52]
Lignin	Vascular plant and algae.	Rich diversity of types and sources; it is a cross-linked complex phenolic polymer, soluble in strong alkaline and sulfite solutions.	[53]
Starch	Seeds, grains, and roots of plants.	It can be used as a thickening agent, stabilizer, disintegrant, binder, etc.	[54]
Xanthan Gum	*Xanthomonas campestris* (Bacteria).	High stability over a wide range of temperatures, pH, and electrolyte concentrations.	[51]
		Better viscosity for use in gels and suspensions.	
Gellan Gum	*Sphingomonas elodea* (Microbial fermentation).	Double helical chain form at low temperatures, presenting single helical chains at high temperatures.	[49,55,56]
		Temperature-dependent structure and viscosity transformation properties, i.e., thermal gelation.	
		Good durability in dry and wet cycles.	
Dextran	*Leuconostoc mesenteroides* and *Streptococcus mutans* (Lactic acid bacteria).	A flexible biopolymer that forms a high density and low permeability in aqueous media.	[57]
β-glucan	Cellulose, bran, and the cell walls of yeasts, fungi, and bacteria.	Water solubility, dispersibility, viscosity, and gelation properties.	[45]
		The natural β-glucan is electrostatically neutral and negatively charged when modified by hydroxyl groups (−OH).	
Curdlan	*Agrobacterium biovars* and *Alcaligenes faecalis* (Pathogenic bacteria).	Elastic but irreversible when heated.	[58]
		Being used as a water reducer in concrete mixtures to prevent the separation of cement aggregates.	
Scleroglucan	*Sclerotium rolfsii* (Fungus).	It has good water retention and thickening properties.	[59]
Casein	Animal proteins.	Hydrophobic, capable of coagulating and forming suspended colloids.	[60]
Gelatin	Animal bones, skin, and tendons.	Soluble in hot water, used as a gelling agent, stabilizer, emulsifier, and thickening agent.	[61,62]

**Table 3:** Interaction mechanism between polymer modifiers and soil.

Polymer Modifier Types	Soil Types	Research Methods	Mechanism of Action	Reference
PAM	Expansive soil and clay	SEM	The gel structure is thin and lean, adhering to the surface of soil particles.	[80]
Polyacrylate	Clay	SEM	Polymer functional groups with the −OH groups of the clay platelets via H-bonding.	[81]
PVAc	Clay	SEM	Filling of voids.	[82]
			Long-chain macromolecules wrap around the surface of the aggregates and interconnect to form elastic and adhesive membrane structures.	
PVA	Soft clay	SEM	Fill in the pores and form larger aggregates.	[74]
PU	Sandy soil	SEM	Sand particles are tightly wrapped by a thin and tough polymer film, which forms a three-dimensional cross-linked network structure among the particles and plays a cementing role.	[18]
MDI	Sand	SEM	Wrap the soil particles and fill the pores.	[78]
Xanthan gum	Silt	SEM	Form sticky hydrogels to coat the soil particles and fill the pores.	[83]
			Form xanthan chains and a “honeycomb”-shaped pore structure.	
	Laterite	FESEM	The gel wraps the soil and forms an interlocking structure with the soil.	[84]
Gellan gum	Silt	SEM	Forming biofilms that produce network structures and gellan micelles that fill soil pores.	[83]
Guar gum	Clay	SEM	Chemical bonding and wrapping bypass.	[85,86]
Lignin	Silt and sandy soil	SEM, XRD, FTIR, and MIP	A flocculent soil structure is produced, and porosity is reduced.	[87,88]
			Cementing material covers the soil and binds and fills the pores	
Persian gum	Kaolinite soil	SEM, SZM, BET, TGA, and PSA	Fill pores, compact structure; reacts with charged clay surfaces through hydrogen bonding and ion interactions.	[52]
			The carboxyl group crosslinks with the negatively charged surface of clay.	
			Forming sticky gels to aggregate soil particles.	
Epoxy resin and aminoamide-based hardener mixtures	Kaolinite clay, bentonite, and cement	SEM and XRD	Epoxy resin provides a gel layer on top of the soil particles, and kaolinite clay does not react in any way with epoxy resin.	[89]
XG-g-PAA	Laterite	FTIR, XRD, TGA, and SEM	The laterite nanoflakes flocculate and disperse homogeneously in the polymer matrix, forming a homogeneous composition.	[90]
			Solid sand specimens containing CSFA show dense contacts in the sand–sand grain transition region, where sand grains bond to each other through CSFA to form a bonding layer.	
			NaA is attached to the XG chain, and the -OH group of the laterite is involved in the polymerization reaction.	
PAA hydrogel	Silty sand	^1^H NMR relaxometry	Releasing gradually into the pores of the soil, the elastomeric gum acts as an adhesive agent.	[91]
			In arid environments, the cementation and friction among soil particles are intensified, thereby enhancing the overall structural stability of the soil.	

FESEM: field-emission scanning electron microscope; FTIR: Fourier-transform infrared; MIP: mercury intrusion porosimetry; XRD: X-ray diffraction; SZM: Stereo Zoom Microscope; BET: Brunauer–Emmet–Teller; TGA: thermal gravimetric analysis; PSA: particle size analysis; ^1^H NMR relaxometry: ^1^H nuclear magnetic resonance relaxometry.

**Table 4:** Comparison of the action mechanisms between synthetic polymer modifiers and biopolymer modifiers.

Polymer Modifier–Soil Interaction Patterns	Synthetic Polymer Modifiers	Biopolymer Modifiers
Filling and adsorption	Hydrophilic functional groups undergo ion-exchange reactions with soil particles, establishing hydrogen bonds and van der Waals forces. The surface layer of these aggregates is coated with long-chain macromolecules.	Modifiers present in the soil matrix undergo gelation, leading to encapsulation, adhesion, and pore-filling effects. Additionally, they exhibit electrostatic interactions that enable adsorption of soil particles.
Pore structure	The formation of a three-dimensional adhesive network structure results in the flocculation of soil particles.	Refining the formation of biological chains and “honeycomb” pore structures within the soil.
Membrane structure	The physical–chemical bond is established, and the modifier is linked to soil particles via chemical bonding, resulting in the formation of an elastic membrane structure.	The colloid–polymer bond is activated, resulting in the formation of a three-dimensional polymer membrane structure and stiffened polymer chains.
Penetrating quality	The enhancement of bonding and reduction in particle spacing is contingent upon the uniform permeability of the modifier solution within the soil.	The infiltration of soil pores is restricted by high viscosity, cohesion, and surface tension. Moreover, the curing effect is significantly influenced by soil particle size.

**Table 5:** Advances in the promotion of vegetation growth through polymer modifiers.

Polymer Modifier Types	Polymer Modifier Dosage	Soil Types	Plant Types	Vegetation Growth Properties	Reference
Acrylamide and potassium acrylate copolymer	10%	Clay	*Caragana korshinskii*	Seed germination rate increased by approximately 244%.	[145]
Hydrogel mixed with a peat-based	1.5%	Clay	*Quercus suber* L.	Survival rate increased by over 20%.	[146]
NBA mixed with SAR (ADNB)	NBA10 g/m^2^ and SAR60 g/m^2^	Silty clay	*Crotalaria pallida*	Plant germination rate increased by 40%, plant height increased by 32.73%, and coverage rate increased by 553.85%.	[147]
Xanthan gum	0.5%	Silt	Ryegrass	28% increase in height.	[83]
Gellan gum	0.5%	Silt	Ryegrass	8% increase in height.	
Guar gum	0.5%	Silt	Ryegrass	4% increase in height.	
Starch	0.5%	In situ soil (Seosan, Korea) and jumunjin sand	Ryegrass	The germination rate increases by about 5%, and the average root length after treatment increases.	[148]
β-glucan	0.5%	Korean red yellow soil	Oats	Increase seed germination rate by 6.6–10.8%.	[17]
Xanthan gum	0.5%	Korean red yellow soil	Oats	Increase seed germination rate by 1.9% to 5.5%.	
M-CMC	1.1%	Sand soil	*Elymus*	Plant biomass increased by 59.65%, plant lodging rate decreased by more than 60%, and drought resistance survival rate increased by more than 80%.	[149]
FA and PAM	10%FA and 0.1%PAM	Sand soil	*A. splendens*	The average height of plants increased by 145%, and the tillers number increased by 2.3 times.	[150]
Potassium polyacrylate polymer	0.08%	Sand soil	*Festuca arundinacea ssp.*	Aerial vegetation biomass doubled in size.	[151]

## Data Availability

Not applicable.
